# A Low-Cost Open Hardware System for Collecting Traffic Data Using Wi-Fi Signal Strength

**DOI:** 10.3390/s18113623

**Published:** 2018-10-25

**Authors:** Shivam Gupta, Albert Hamzin, Auriol Degbelo

**Affiliations:** Institute For Geoinformatics, Westfälische Wilhelms-Universität, 48149 Münster, Germany; albert.hamzin@uni-muenster.de (A.H.); degbelo@uni-muenster.de (A.D.)

**Keywords:** traffic counter, WiFi signals, open hardware, traffic monitoring, low cost sensors, smart cities

## Abstract

Road traffic and its impacts affect various aspects of wellbeing with safety, congestion and pollution being of significant concern in cities. Although there have been a large number of works done in the field of traffic data collection, there are several barriers which restrict the collection of traffic data at higher resolution in the cities. Installation and maintenance costs can act as a disincentive to use existing methods (e.g., loop detectors, video analysis) at a large scale and hence limit their deployment to only a few roads of the city. This paper presents an approach for vehicle counting using a low cost, simple and easily installable system. In the proposed system, vehicles (i.e., bicycles, cars, trucks) are counted by means of variations in the WiFi signals. Experiments with the developed hardware in two different scenarios—low traffic (i.e., 400 objects) and heavy traffic roads (i.e., 1000 objects)—demonstrate its ability to detect cars and trucks. The system can be used to provide estimates of vehicle numbers for streets not covered by official traffic monitoring techniques in future smart cities.

## 1. Introduction

The rapid escalation of the population in urban spaces accompanied by increasing demands for mobility in cities [1], leads to substantial challenges in city planning. Increasing demands on mobility lead to growing traffic on the road, inducing suffering for citizens concerning the reduction of travel efficiency, increase in fuel consumption and health hazards from air and noise pollution caused by vehicles. In addition to being a significant source of air pollution in cities, road traffic exposes a large number of people to high daytime noise levels [2,3]. Road traffic also leads to anthropogenic heat that together with reradiation effects from urban spaces can increase urban space temperature, resulting in urban heat islands (UHI) [4]. Hence, it is of great importance to monitor the complex interplay of the road network and traffic conditions for better of sensing Quality of Life (QoL) in future smart cities.

Road traffic is one of the major sources of air pollution in cities [5]. It is a significant anthropogenic source of $NO_{x}$ [6], particulate matter (PM) and other harmful pollutants which impact human health [5]. Exposure to road traffic induced air pollution can lead to various health impairment for the current as well as the future generation. Multiple studies demonstrated the association of traffic generated air pollution to different heart-related disease in adults as well as for pregnant women [7,8]. Traffic data is one of the critical input variables for air pollution modelling approaches [9,10].

For many years, various approaches have been devised to monitor air pollution at a higher resolution in the city [10]. High-resolution monitoring approaches require input parameters also at a higher resolution. However, most studies used traffic models and simulation to represent traffic data because the traffic monitoring datasets are usually available for a very limited number of roads in the cities, hence limiting the possibilities to model air pollution at higher resolution in the city.

Traffic data collection was traditionally performed by using manual processes or with the application of inductive loops at certain locations [11]. The inductive loops for traffic monitoring became standards in many jurisdictions and are widely used till date [12]. Various other conventional traffic monitoring approaches include passive infrared devices, Doppler and radar microwave sensors, acoustic detectors, magnetic strips, Piezoelectric sensors, Pneumatic road tube counting devices and video vehicle detection. However, these approaches inherit certain limitations (notably installation and maintenance costs) making them hard to deploy for detailed data collection with better spatial coverage in cities.

The vision of "smart cities" was proposed to address particular problems caused by urbanisation and to promote sustainable urban development in cities. This vision relies on the efficient application of information and communication technology (ICT) for sensing, analysing, integrating critical information which can support efficient operation and development of cities. Improved traffic control was identified in [13] as one of the possible benefits of advanced sensing in smart cities. Taking into account the emergence and rapid growth of the Internet of Things (IoT) and analytical tools, future cities may be able to enhance the execution and connectivity of urban services, reduce costs and operate on better resource management. The recent advancements in microelectronics, telecommunications and data analysis domains have led to the growing adoption of smart devices. With the application of these smart devices, it is possible to overcome detailed traffic data collection challenges. Extending the deployment of the low-cost IoT devices in a distributed model like crowdsourcing can support well-spread data collection in cities. Altogether, the recent developments in low-cost hardware and support from the crowd can help gather data, which can support transport planning and urban health risk assessment for cities.

The benefit of involving citizens for crowdsourcing traffic data is the ability to provide real-time information about traffic; better situation awareness especially in areas where official traffic monitoring systems are not installed (e.g., rural areas and residential areas); and better spatial coverage. For cities administrations which are not purchasing the data from various commercial third-party traffic data providers (such as TomTom, or HERE), crowdsourcing traffic data can be a useful and low-cost data source. In general (and as indicated by Degbelo et al. [14]), without engaging citizens in the creation and sharing of data for the provision city services, a city may only be halfway open and smart. Providing means which lower participation barriers to citizens (e.g., low cost sensors) is thus critical to citizen empowerment in future smart cities.

In this paper, we present a novel system based on open-source hardware that has the potential to benefit traffic monitoring technologies because of its low-cost, privacy-preserving, ease of application and potential to large-scale deployment. Because the system is low-cost (less than $50), it can be used to involve citizens in WiFi-based traffic data crowdsourcing projects, and this way expand traffic data collection to streets currently not covered by conventional traffic monitoring techniques. Section 2 briefly discusses previous work done related to the topic. Section 3 describes technologies and the sensor used for the traffic monitoring and presents the algorithm used to infer the results from the proposed system. In Section 4, we present the results we obtained concerning the performance of the proposed system in the real world, using two different scenarios. Section 5 discusses the results we obtained, as well as the applications and limitations of the proposed system. Section 6 and Section 7 present the future work and conclude the work respectively.

## 2. Related Work

Traffic monitoring in cities involves, among other things, estimating the number of vehicles on the road. The vehicles are tracked from point to point along the road for their information. A traffic monitoring station is used to measure traffic parameters such as vehicle count, speed and occupancy at a specific location. The measurement at monitoring station locations is to be representative of the traffic on the road. Generally, vehicle detection and traffic surveillance involve a network of devices deployed at various roads of the cities. This section presents a brief overview of the various types of technologies and devices used for traffic surveillance with a particular focus on low-cost sensors, as it is also the primary focus of this paper. Furthermore, this section also discusses in brief privacy-related concerns while deploying traffic surveillance systems.

### 2.1. Traffic Monitoring Techniques

Traffic monitoring is a vital, yet challenging since to built traffic density maps traffic parameters such as vehicle count, location, speed and follow of vehicles are required. One of the essential requirement for efficient traffic systems is the reliable and real-time traffic data collecting network of devices to facilitate instantaneous decision-making. The technologies used in the devices for vehicle detection and traffic surveillance can be classified into the following five categories (See also the list of traffic sensing technologies frequently employed in traffic surveillance for data collection provided in [15]):

1. Intrusive devices
2. Non-intrusive devices
3. Off-roadways devices
4. Sensor combinations devices
5. Relatively low-cost devices

#### 2.1.1. Intrusive Devices

Intrusive devices are installed directly into the pavement surface by creating saw-cuts or holes in the road surface, by burrowing them under the surface, or by anchoring them directly into the pavement surface. Devices such as inductive loops (IDL), magnetic detectors, micro-loop probes, pneumatic road tubes, piezoelectric and other weigh-in-motion devices are considered as intrusive devices. These devices are highly accurate for vehicle detection (>97%) [16]. However, a major drawback concerning the use of the intrusive devices is the disruption of traffic caused for installation, repair and failure associated with installation in poor surfaces and use of substandard installation procedures [17]. These devices are also expensive, large and consume much power which limits their implementation for better spatial coverage in cities [18]. Resurfacing and repair tasks on the roads can also create the need for reinstallation of these devices. The safety of workers, those who are deploying these devices has also been a matter of concern [18].

#### 2.1.2. Non-Intrusive Devices

Non-intrusive devices are a more reliable and cost-effective vehicle detection and surveillance devices than intrusive devices. They can be easily installed, maintained with safety with minimal disruption to traffic flow, and can provide traffic data with similar accuracy to that of inductive loop detectors [17]. Non-intrusive devices include technologies such as video image processing, microwave radar, laser radar, passive infrared, ultrasonic, passive acoustic array, in which devices are mounted overhead on roadways or roadsides. These devices are capable of measuring vehicle count, presence, and passage on the road. Some devices also have the potential to provide vehicle speed, vehicle classification, and multiple-lane, multiple-detection zone coverage [19,20]. However, the devices fail to perform in certain environmental conditions. For instance, infrared devices can be affected by fog, and temperature change, video image processing devices detection efficiency can be hampered by weather conditions, shadows, vehicle projection into adjacent lanes, day-night transitions, vehicle/road contrasts and water salt grime or cowebs on camera lens [17]. The relatively high deployment and maintenance cost of the aforementioned technologies limits the large-scale integration of these devices into the traffic surveillance systems [17].

#### 2.1.3. Off-Roadways Devices

These devices use the technologies that do not require any hardware deployment under the pavement or mounted overhead/roadside. The devices enable traffic monitoring via aircraft or satellite, as well as by probing the vehicles equipped with Automatic vehicle identification (AVI), Global Positioning System (GPS) and mobile phones [21]. These technologies can help in enabling the high percentage of roads coverage. However, privacy concerns and other technology-specific limitations restrict their application [22].

#### 2.1.4. Sensor Combinations Devices

Due to certain limitations of individual technologies, various studies suggested the application of the off-roadway devices together with more than one technology to monitor traffic flow on the road. Applications include the combination of passive infrared with ultrasound and Doppler microwave radar, which enhanced the accuracy for vehicle detection in queues and counting them along with their height and distance discrimination [17]. Nevertheless, the cost of deployment and its complexity limit the well-spread deployment of a network in the cities.

#### 2.1.5. Relatively Low-Cost Devices

The scalability and availability of traffic monitoring systems are essential for efficient and reliable, real-time traffic monitoring [23]. Devices like Magnetometer (MAG) have been found to serve the requirement [24] but the maintenance and installation cost along with limitations with radar detectors impact the performance [24]. Low-cost, portable, and easy-to-install technologies are desired to supplement existing data sources for efficient, detailed traffic monitoring in the cities [18]. The availability of new low-cost and miniaturised hardware platforms has enabled the idea of developing advanced and pervasive image-based devices, which can help in vehicle counting and traffic surveillance. Till date, several approaches have been proposed to investigate the feasibility of vehicle detection and traffic surveillance using low-cost sensors.

A method which uses continuous-wave radar was presented by Fang et al. [25]. The method uses an antenna, a microwave radio front, the analogue signal amplifier and the digital signal processor (DSP) for vehicle detection. A computer vision application enabling vehicles monitoring by using low-cost and low-complexity devices was proposed by Salvadori et al. [26]. Recently, WiFi signal-based approaches were used for assessing human activity recognition [27,28,29,30] suggesting possible additional applications of the WiFi technology other than providing easy internet access. Approaches based on channel state information (CSI) [31], link quality indicator (LQI) [32], packet loss rate [32], and received signal strength indicator were proposed for vehicle detection using radio waves [33,34]. However, the methods mentioned above are not well suited for crowdsourcing applications because of their expensive specialised hardware (laptop specific WiFi cards and modules) or energy data transfer requirements. With regards to computer vision applications, low-cost devices constrain its computational capabilities and available onboard memory, making it unfeasible for effective implementation [26]. Approaches using Bluetooth low-energy beacons and smartphones were also proposed for enabling crowdsourcing of traffic data with low-cost devices [35]. However, use of a smartphone by users at the roadside for traffic monitoring seems a bit impractical. Moreover, gathering data by using smartphones or video analysis based system also impose the threat on the privacy of the commuters on the road.

Table 1 presents an overview of all the existing technologies we discussed with their respective advantages and disadvantages.

### 2.2. Privacy and Traffic Monitoring

Traffic surveillance and vehicle detection systems are promising approaches which are cyber-physical by nature. These cyber-enabled systems face various security and privacy preserving challenges [37]. If the vehicles’ location privacy cannot be preserved, commuters may object to the process of being monitored in such systems. If devices which can collect privacy sensitive data is handed over for crowdsourcing, it could be a bigger threat to the society. Hence, the devices should be capable of appropriately protecting the privacy of the vehicle and the commuters on the road [38,39]. With the wider application of computer vision technologies for traffic monitoring, it is important to realise the impact of the approach on the commuters. Privacy and security is one side effect caused by the application of computer vision methods [40]. Several approaches are proposed to solve the privacy problem in traffic monitoring approaches. Lu et al. [41] proposed conditional privacy-preserving protocol, Lin et al. [42] presented conditional privacy and group signature building techniques. The techniques for dealing with unlinkable pseudo-ID were also proposed by Raya and Hubaux [43]. However, these are not implemented for the latest transition of approaches to low-cost devices. Since the primary focus of the paper is to enable counting and identification of vehicles on the road using low-cost devices for crowdsourcing, it is easy to have a system which only enables the measurement of the specific parameter and discarding sensitive data collection on the origin itself.

The system proposed in this paper uses a low-cost open hardware which uses the WiFi signal, which is commonly available at all locations these days to traffic data. The approach has the potential to overcome various limitations existing in current systems discussed above and also preserve the privacy of the commuters.

## 3. Materials and Methods

### 3.1. System Design

**Figure 1:**
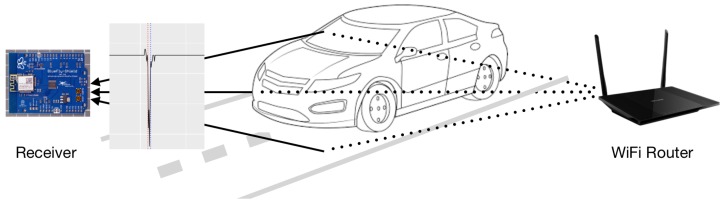
Illustration of deployment plan for the proposed hardware system.

In this section, we present the proposed vehicle detection and counting system using the received signal strength indicator (RSSI) data produced from the router and collected by the receiver, a low-cost open hardware system. The deployment plan for the proposed traffic monitoring system is illustrated in Figure 1.

#### 3.1.1. Receiver

The receiver hardware system was developed using an Arduino Uno R3 single-board microcontroller mounted with a BlueFly-Shield (ATWINC1500) and an SD card shield V3.0 (Model: INT106D1P). The hardware system receives the WiFi signal transmitted from a TP-Link router for our study case.

Arduino Uno R3 is a simple microcontroller with simpler software structure. The Arduino works according to the modular principle with the simple procedure to add components. We mounted two components to capture and store WiFi signals. The first component was the WiFi shield (ATWINC1500), which receive the WiFi signal from connection created via IEEE 802.11n standard and works with the encryption type WPA2. The second component was an SD card shield V3.0 (Model: INT106D1P) that stores the WiFi strength in decibel (dB) and time in milliseconds (ms). The code was written in the integrated development environment (IDE) which runs on the chip. No firmware, interpreter or operating system was involved in the process, which makes the whole procedure easier to implement and also limit the noise when receiving the signal.

#### 3.1.2. Transmitter

For the transmission of the WiFi signals in the study, we used the router from TP-LINK (Model: TL-WDR3600) with features like dual-band with 2.4 GHz & 5 GHz bands and an Atheros Chip. We installed OpenWrt (https://openwrt.org/ (last accessed: 21 August 2018)), an open source project based on Linux with the ability to allow specific changes in physical settings of the radio hardware such as operating frequency, transmit power and encryption. OpenWrt runs on a router only with necessary scripts. These scripts/activities can be enabled or disabled at any time. As a result, the router works in a simple version without heavy background activities. The following settings were used during our study:

- Band: 2.4 GHz
- Standard: Wireless-N
- Width: 20 MHz
- Encryption: mixed WPA/WPA2 PSK (CCMP)
- Bitrate: 144.4 Mbit/s
- Time lapse in the data capture: 100 ms
- Transmission time: 10 ms
- Packet size: 88.5 Bytes

The 2.4 GHz band was used in our study instead of 5 GHz because of the wide range and the compatibility with the receiver hardware system. It should be noted that the introduced system structure, which includes low-cost open hardware system has, to the best of the authors’ knowledge, not been considered in the literature. The transmitter and the receiver were installed at different (opposite) sides of the road, using WiFi signals to communicate with each other. The interference caused by the passing vehicle to the communication pathway between the two devices is measured by the receiver which is then stored in the SD card mounted present in the device. Distinct patterns of change in RSSI are observed when vehicles pass, which is captured and further used for analysis to count and detect the type of vehicle on the road.

### 3.2. System Implementation

For the implementation of the proposed system, we deployed the receiver and the transmitter on the roadside as shown in Figure 1. The proposed system was evaluated for two different scenarios: (1) low traffic road and (2) heavy traffic road. The low traffic road in our study is the road called Heisenbergstraße, which can also be considered as the local road with fewer cars. The heavy traffic road we used as an environment in our study is Steinfurter Straße, which is one of the busiest roads in the city. Both roads are situated in the city of Muenster, Germany. More than 500 objects for Scenario 1 and more than 2000 objects were recorded for Scenario 2 during the real-world field data collection. The distance between transmitter and receiver for Scenario 1 and 2 were 7.5 m and 12.5 m, respectively.

**Figure 2:**
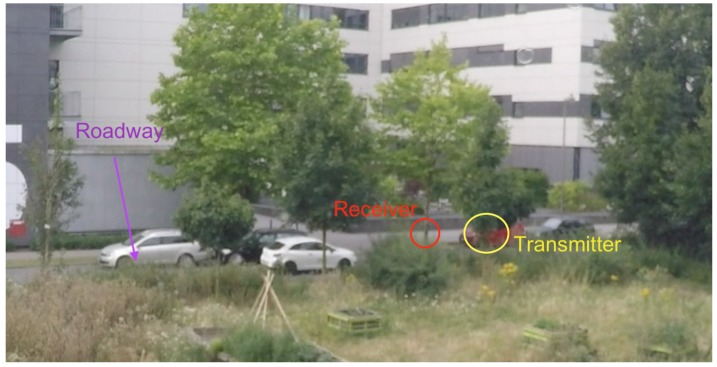
Experimental setup: Scenario 1 (Low traffic road: Heisenbergstraße).

**Figure 3:**
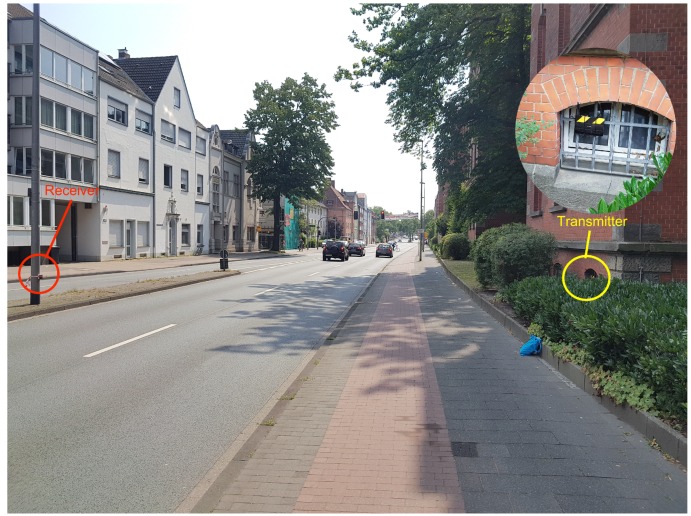
Experimental setup: Scenario 2 (Heavy traffic road: Steinfurter Straße).

To collect the ground truth data we deployed GoPro HERO4 camera that collect video data. Figure 2 and Figure 3 depicts the setup of the proposed system in the two different scenarios under consideration. The low traffic scenario was considered in the study to understand the accuracy of the system under limited vehicles with complexity caused by bicycles, pedestrians and other objects on the road. In contrast, the heavy traffic scenario was considered to assess the performance of the proposed system with complications created by the large number and frequency of vehicles on a road with two lanes. The speed of the vehicles were not assessed during the study, but the speed limit for the roads under consideration were 30 kmph (Scenario 1) and 50 kmph (Scenario 2). Based on the data collected during the study time (and counted with the web application presented below) The hourly average frequency of vehicles passing from both the roads are:

1. Heisenbergstrasse: 103 Cars/hour and 286 Bicycles/hour
2. Steinfurter Straße: 32 Trucks/hour, 684 Cars/hour and 50 Bicylce/hour

**Figure 4:**
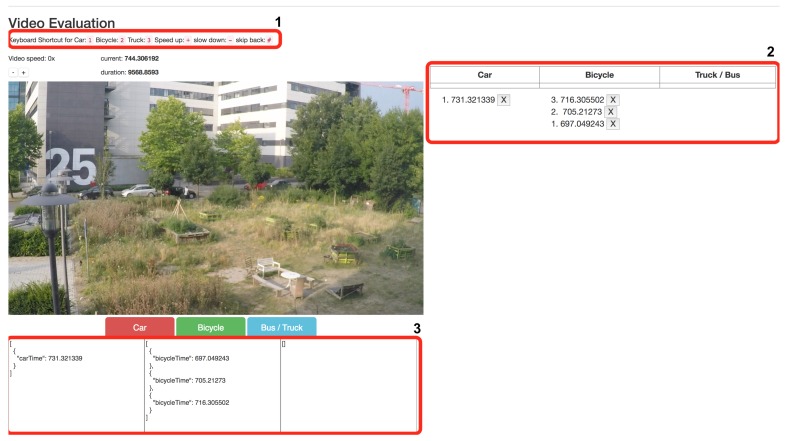
Illustration of the web-application developed for ground-truth data video stream analysis.

To evaluate the accuracy of the proposed system, it is important to prepare the **ground-truth** data video stream at the same scale as the data collected by the receiver. We have also developed a web application which enables the processing of video streams and register the type, number and time stamp of the object on the road. Figure 4 illustrates the developed web-application. The user of the web-application needs to watch the video and manually select the type of object (car, truck, bike, pedestrian and so on), the system then takes the timestamp and count the total number of specific type of objects identified automatically. The input to select the type of object can be done by clicking on the web application’s buttons or by using keyboard shortcuts. The outputs of the video data analysis are stored as JSON files, with each file representing a vehicle type under study with the timestamp in milliseconds, same time scale as that of the dataset generated by the proposed system. During the study, the objects were mainly counted by one researcher. However, a second researcher independently redid the count on 20% of video data for Scenario 1 to confirm the number of objects counted. The Cohen’s kappa coefficient was calculated to assess the agreement between the two, and the result indicates a very high overall inter-rater agreement (0.8 for bicycles and 1 for cars). The web application and the scripts used in the study can be customised as per the requirement and is available online (see [44]) as an open-source tool under Creative Commons licence.

## 4. Results

**Figure 5:**
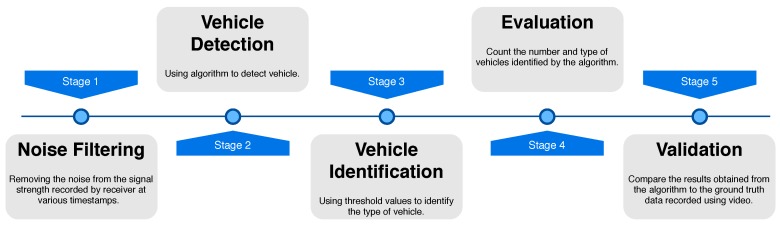
Overall flow of analysis.

This section evaluates the performance of the proposed hardware device. The RSSI changes detected by the open-hardware was stored in an SD-card. The stored data stream was then processed to evaluate the performance. The data processing involves multiple steps as shown in Figure 5.

### 4.1. Noise Filtering

Whenever an object interferes with the communication path of receiver and transmitter, the RSSI fluctuates. The pattern in fluctuation can be a useful indicator to detect the vehicle on the road. In order to isolate the RSSI fluctuation patterns in the data stream, during prepossessing of data, we removed all fluctuations of strength less than or equal to 2 dB, as noise. The deletion of the 2 dB signal was conducted to ignore the usual noises in the WiFi signals transmission and on the receiving end. The leftover data stream was used in the subsequent steps to detect vehicles using algorithms based on certain parameters of the RSSI fluctuation patterns.

### 4.2. Vehicle Detection

To detect the vehicle using RSSI fluctuations, an algorithm was used to detect the patters in the data stream collected by the proposed device. Two parameters, namely: (i) Maximum RSSI value and (ii) Time window were used in the algorithm to characterise the signal patterns from the stream of data. These two parameter were then further used in machine learning algorithm; k-Nearest Neighbour for vehicle identification.

#### 4.2.1. Maximum RSSI Value

**Figure 6:**
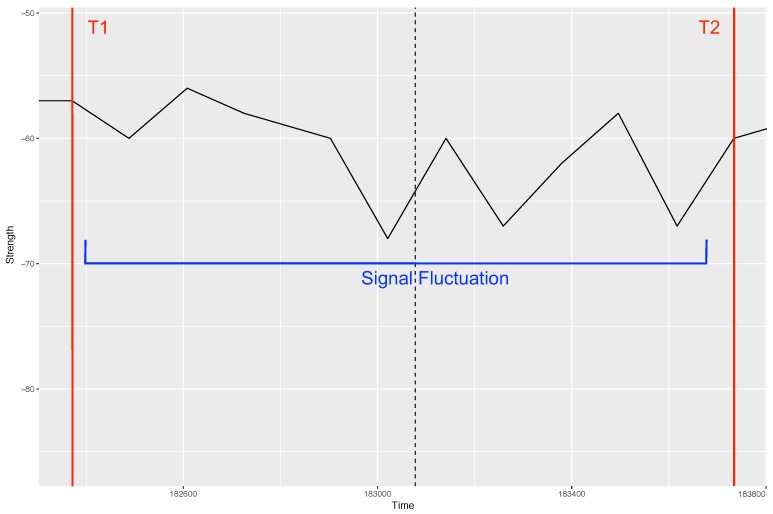
Illustration of time window and associated signal fluctuation pattern identification (Units: Time = Milliseconds, Strength = dBm).

To recognise patterns which can represent vehicle movement, the data stream after noise filtering was analysed. In the algorithm, we identify a pattern in signal fluctuation by comparing the signal strength at each time stamp with the preceding and subsequent time stamp of the data. During comparison, if the signal strength change is positive for the consecutive three time stamps we start recording the initial time (say $T_{1}$) and the end time (say $T_{2}$) where the significant signal fluctuation stops (difference between signal reaches 0). The $T_{1}$ and $T_{2}$ recorded for each fluctuation pattern identified provides the time window along with the respective signal strength, which translates several curves of the time window into a single integer. The maximum strength value for each time window acts as an indicator of the presence of certain vehicle (see Figure 6).

After the identification of the time window and the signal strength associated, the maximum signal strength change for each time window was computed. This maximum change in signal strength for a given time window can help in identifying the type of vehicle. However, threshold values are required to define the rule based on which the algorithm can further identify the different types of objects.

#### 4.2.2. Time Window

As can be inferred from Figure 6, the time window is used for recording the patterns in the collected data stream. We also used the the length of time window, i.e., difference between $T_{2}$ and $T_{1}$ to identify the type of vehicle (Equation (1)). The intuition behind this is that different vehicles can produce disturbances of varied temporal length. For instance, if the fluctuation continues for a long time window, there is a possibility of having a long vehicle between the transmitter and receiver communication path. Here also, deciding the threshold for the time window size is important to characterise different sizes of vehicles that can help in identifying the type of vehicle.

(1) $$Time\;window\;size=T_{2}-T_{1}$$

**Figure 7:**
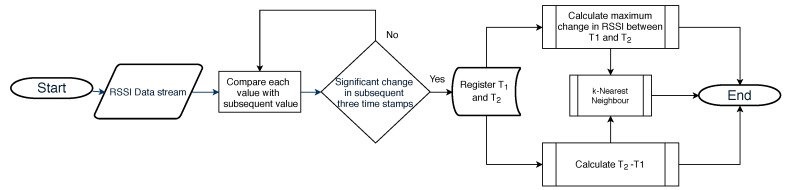
Flow chart of algorithm for vehicle detection.

Figure 7 below represents the overall flow of the algorithm:

### 4.3. Vehicle Identification

In the study, we used the data from the hardware device to identify three types of vehicles, namely bicycles, cars, and trucks. As discussed previously, defining thresholds is necessary to characterise the type of vehicle. In our study, we defined the threshold using the summary statistics of both, the maximum RSSI fluctuation value and time window size.

#### 4.3.1. Using Two Parameter Values

**Figure 8:**
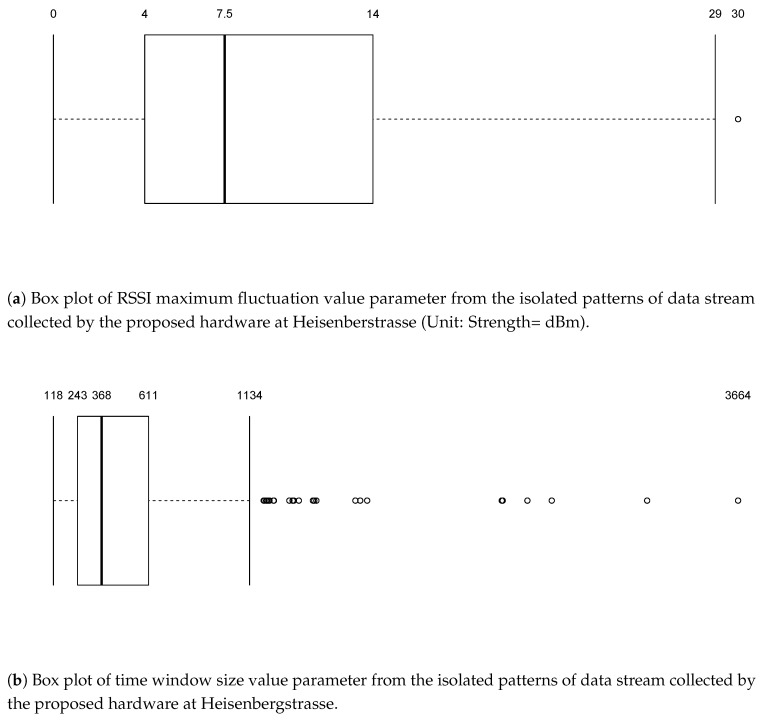
Parameters summary statistics for Heisenbergstrasse.

**Figure 9:**
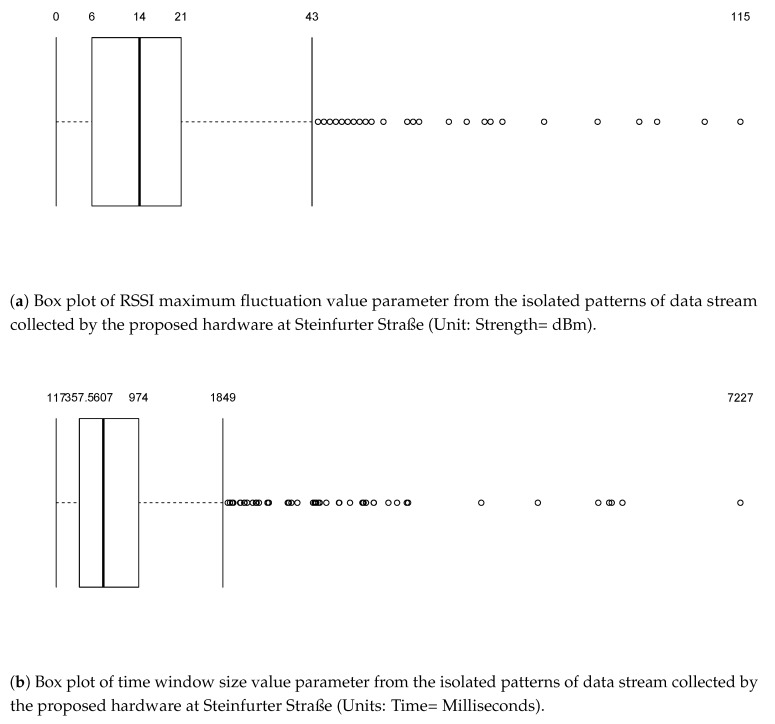
Parameters summary statistics for Steinfurter Straße.

Figure 8 and Figure 9 present the summary statistics we observed for the data calculated using the above-mentioned algorithm for Heisenbergstrasse and Steinfurter Straße. As the figures show, there are lots of outliers at a time, illustrating a lack of regularity in the detection of signal fluctuations using the device. This was not entirely unexpected, given the low cost of the receiver hardware.

Based on the summary statistics (and visual inspection of the data), we defined the most plausible thresholds for vehicle identification. The threshold was calculated for both the scenarios separately because of the infrastructure available around the study area. There are two possible reasons for this: (1) the distance between the transmitter and receiver was different in the two scenarios, as said in Section 3; and (2) the use of multi-directional transmitter, which could have induced different wave propagation in the different environments where the device was deployed. For Scenario 1 the hardware setup was deployed in an open space (Figure 2), while the setup was deployed in a partially close environment in Scenario 2, as can be seen from Figure 3. The walls around the study area and the distance between the transmitter and receiver could have contributed to noise caused by reflection and diffraction of WiFi signals. Threshold values were chosen so that they coincide with the values of the quartiles. Table 2 and Table 3 present the threshold rules we used for vehicle detection in each of the two test scenarios under consideration.

#### 4.3.2. Using Machine Learning: k-Nearest Neighbour (kNN)

After considering the sensitivity of the proposed low-cost device for vehicle detection, we performed the preliminary investigation to identify the potential of machine learning method for vehicle type identification. We used the values obtained for the two parameters calculated in the previous steps for the machine learning method kNN. A dataset of RSSI values and its associated two parameter values were used to train the algorithm and to get the two value parameter value mapped for each vehicle type. The training dataset of n-dimension space is stored for two scenarios. Given a target values of the two parameters, the method determines the training dataset to find the k nearest matching data records, to find the target type of vehicle trained in the training process. To check the performance of the classification process a two-fold cross-validation was performed. The k-value for the complete dataset was increased to reduce the impact of noise in the kNN process. The training for the classification process was done in supervised manner with following labels for two scenarios under consideration:

- Scenario 1: Three labels - *Bicycle*, *Car* and *No vehicle*
- Scenario 2: Four labels - *Bicycle*, *Car*, *Truck* and *No vehicle*

### 4.4. Evaluation

After defining the threshold rules, the algorithm is capable of identifying the vehicle type. The vehicle identification finally helps in counting the number of vehicles using the particular road. We compared the accuracy of the algorithm with the ground truth data we measured after interpreting video recording into JSON files for each test case scenario. Table 4 and Table 5 present the comparison between the ground truth data from video and vehicle classification using the algorithm parameters we used in the study. 

**Scenario 1: Heisenbergstrasse**

**Scenario 2: Steinfurter Straße**

### 4.5. Validation: Precision, Recall and F Measure

To understand the reliability and performance measures of the proposed device in the two different test case scenarios, we computed the precision, recall and F measure. The calculation for precision, recall and F measure was done using the following equations:

(2) $$Precision=\frac{tp}{tp+fp}$$

(3) $$Recall=\frac{tp}{tp+fn}$$

(4) $$F\;measure=2\times \frac{Precision\times Recall}{Precision+Recall}$$

where, the sum of tp (True Positive) and fp (False Positive) is the total number of Vehicles detected using the parameters of the algorithm sub-steps, tp is the number of vehicles actually overlapping (temporally) with vehicles in the ground truth data. In Equation (3), the sum of tp and fn (False Negative) is the total number of objects detected in the video. Table 6 and Table 7 summarise the results for each object in both test case scenarios. 

**For Heisenbergstrasse:**

**For Steinfurter Straße:**

## 5. Discussion

This study has explored the potential of using low-cost open hardware for WiFi-based vehicle count. Given existing initiatives to increase free WiFi hotspots in cities (e.g., European Commission [45]), there is a need for techniques which take advantage of WiFi availability for traffic monitoring purposes. The advantage of low-cost sensors over expensive, highly performant sensors is that they can be bought by a large number of citizens. As a result, they can be deployed on any road of the city, enabling data collection about traffic at places currently uncovered by official traffic monitoring techniques. It can be inferred from the results that the proposed system is capable of identifying certain types of vehicles on the road with great reliability. The classification accuracy on the low traffic road suggests the ability of the system to identify objects like bicycles and cars with higher accuracy. However, the ability of the system to detect a comparatively small object such as bicycles for the busy roads (Scenario 2) is low but can characterise cars and trucks with high accuracy. That is, the proposed system is useful for collecting data about cars and trucks in the residential areas, where traffic data is not generally collected.

Looking at Table 6 and Table 7, one can conclude that the system *can* be used to count vehicles, as can be inferred from the values for precision/recall after taking into account the machine learning method. However, due care must be exercised in interpreting the kNN based results as the findings are in a preliminary stage and were investigated to comprehend the potential of machine learning methods with the proposed low-cost hardware device. Counting based on RSSI and time window alone is not very efficient, as one can conclude from the recall values (roughly around 0.33) for cars/trucks in both cases. The high improvement in precision/recall resultant from the use of kNN comes at the price, namely, the *price of training the algorithm for each new context*. If this expertise is not available, RSSI and time-based techniques could be used as alternatives. They do not require training for the algorithm and rely on thresholds determined systematically based on summary statistics. The recall values being around 0.33 = 1/3 in the cases of RSSI/time-based detection suggests that RSSI-based and time window-based counts can be used to make *rough estimates* about the number of cars/trucks which have been on the street during a time period. For instance, if one would detect 2 cars/trucks with the code, there were roughly 6 cars/trucks on the street; if one has 1000 cars/trucks in the code, roughly 3000 cars/trucks were available in reality, and so on if only threshold values are used without kNN process. Overall, there is evidence from Table 6 and Table 7 that the whole approach of using low-cost sensors to count vehicles based on WiFi signals is workable and pertinent on the road towards smarter cities.

One thing worth mentioning regarding RSSI and time window based identification is that though the precision/recall end up being relatively low, the numbers of vehicles identified by the proposed hardware in Table 4 and Table 5 are actually quite close to the numbers of the vehicles in the videos (which suggests a good overall accuracy for the technique). The precision/recall values computed subsequently using only the thresholds (Table 6 and Table 7) led to much lower numbers when it comes to the overall performance. This could be due to the process of computing precision/recall values during the work. An object from the code was said to correspond to an object in the video *if and only if* the time $T_{0}$ of the video object was within the time window of the signal fluctuation (see Figure 6). Since the units of measurements were all in milliseconds, and the times $T_{0}$ of the video objects were manually identified, there are chances that overlaps between the two were missed by the algorithm. Relaxing the ‘if and only if’ constraint could have led to different values of precision/recall. Furthermore, the precision of bicycle detection (Table 7) even after using the kNN method for Scenario 2 was very low. This low precision can be because of the high rate of fluctuations in signals due to heavy traffic on the road and the system setup where signal reflection are high, leading to several noisy signals which algorithm interpret as bicycles (as RSSI change by bicycles is small). Hence, the method’s performance to detect small vehicles on the busy roads is limited.

We believe that our system can help in overcoming limitations imposed by traditional existing methods. The proposed system does not require any installation within the road surface or overhead that can lead to hindrance in the normal traffic flow. This helps in overcoming the limitations imposed by various intrusive methods as discussed by Mimbela and Klein [17]. The proposed system in its current state only stores the WiFi signal data with particular time stamp value, making the data flow easy and less in volume, which helps in overcoming the barriers imposed by low-cost computer vision-based traffic detection methods. Overall the proposed system addresses limitations (i.e., cost, privacy concerns, high maintenance requirements, weather and light effect) imposed by the various methods discussed in Section 2. Since the system uses WiFi signals, it is tolerant to weather conditions, such as rain or thunderstorms, which is a significant advantage over various computer vision-based devices, ultrasonic devices and related technologies. The proposed approach can also be considered as an excellent fit to overcome performance issues, such as the effect of weather and light condition, pointed out by Balid et al. [18] for vision-based or radar-based low-cost devices. The system only uses RSSI of WiFi signal to access the vehicles on the road, which can be considered as the simpler version of the approach suggested by Won et al. [31]. Lastly, the technique proposed does not use any sensitive data, making it privacy-friendly during traffic data collection. Table 8 presents an assessment of various technologies with respect to the dimensions considered in the present paper, namely: high spatial coverage, insensitivity to weather conditions, low-cost, compactness, usefulness for crowdsourcing and the capability to preserve the privacy of the commuters. ‘High spatial coverage’ concerns the ease of using the technology for widespread, large-scale deployment in a study area. ‘Compactness’ defines the aspect of the compact size of the hardware device, which can help in easy deployment of the system; ‘Relevance to crowdsourcing’ refers to the pertinence of the technology to crowdsourcing scenarios. For example, the Bluetooth technology-based solution proposed by Lewandowski et al. [35] for traffic monitoring can be useful for crowdsourcing, but the aspect concerning the deployment of mobile phones at the side of roads seems impractical in our view. Hence we characterise it as not so relevant for crowdsourcing in the table.

The system is also subject to some limitations, one major one being its inability to differentiate between noise and objects in a few instances. This can be because of the low power of the WiFi signals. During the study, the system represented slow-moving pedestrians and fast-moving cars as the same object, which lead to a few false vehicle identifications or sometimes even missing the vehicle. The proposed method also has issues concerning the identification of small objects like bicycles on busy roads. The low-cost sensor also showed unusual behaviours at certain temperatures because of heating or ambient temperatures, which could be warrant a closer, more systematic look in future studies. Sometimes the sensor behaves unexpectedly leading to no signals or very high peaks in data. These limitations are inherent to any low-cost sensing device. Another limitation could the fluctuating tolerance of WiFi receiver to specific signal strength, making some of the legitimate peaks of objects as noise in the dataset.

Another critical factor is the identification of the time window for the calculation of the two parameters used in the study. The start and end value identification may be different for different roads, but also significant fluctuations in the signal may lead to selection of different T values. Furthermore, various surrounding situations like the gathering of people or proximity to vehicle parking can trigger changes in values of signal at any point during the deployment. These factors can change the observation value fluctuation to other extreme values, changing the overall start and end time value calculations for time window that can effect the vehicle identification accuracy.

In all, the proposed system is capable of overcoming some limitations which exist in various traffic monitoring methods. It is also useful in addressing the privacy concerns, making the data collection process easy and fast for real-time traffic monitoring. The system’s unresponsiveness to weather condition and luminescence related conditions make this method more advantageous than other sophisticated methods such as vision-based technologies or infrared technologies. The very low-cost of the whole infrastructure ($50) can facilitate the traffic data collection using a large number of devices, enabling better spatial spread for detailed city-level traffic data. The easy deployment capability and operation with generally available WiFi signals make the system useful for crowdsourcing, which can encourage citizen participation and open data collection for open smart cities initiatives.

## 6. Outlook

The proposed system currently uses summary statistics for threshold identification for vehicle type identification, if only parameter values are used. We extended the approach using kNN machine learning process. Since the machine learning-based investigation presented in this paper is at an early stage, extending it to use other sophisticated machine learning procedures for vehicle recognition using the raw signal stream directly from the receiver, may help in overcoming some limitations imposed by segregating data based on time window values. In addition, the technique has been only focused on measuring the potential of the low-cost sensor device to detect one vehicle at a time. Extending it so that it can identify aggregated peaks of two objects by using various statistical approaches could also be worth considering in future work. The proposed method also has not considered the aspect of congestion on the road and its impact on the vehicle identification process; future work could incorporate these two aspects in the identification process. Another future work could be to improve the performance of the proposed system by developing new modules which can help in differentiating various small and big object with speed parameters. Extending the current method with approaches suggested in [31,32] for low-cost open hardware is also another possible direction for future research.

The accuracy of the proposed method was evaluated by using the video data which is converted to the same scale as data from the receiver by manually watching the video. This process is error-prone, as the time ascribed by the human being while counting the video object may not be the exact original time at which the object crossed the WiFi infrastructure. Future work may consider automating the current manual process with the application of vision-based analysis, such as using old phone cameras in combination with proposed hardware. The low-resolution camera from used phones or other electronic wastes with just enough resolution to differentiate between small and big objects on the road can help with the real-time feedback mechanism. Integrating the low-cost video devices data with WiFi receiver data stream can improve the overall accuracy of vehicle detection and counting.

## 7. Conclusions

In this paper, we presented a WiFi RSSI-based traffic monitoring system using low-cost open hardware that is capable of providing essential functionalities for vehicle detection and counting. Real-world tests suggest that the proposed method is capable of detecting car and bicycles in low traffic and car and trucks in heavy traffic scenarios. The proposed system relies on the use of summary statistics to come up with thresholds for vehicle type identification, which once extended with a kNN machine learning process, produce promising results. The proposed system is tolerant to weather conditions and also helps in preserving the privacy of the commuters by not using any sensitive data for vehicle identification. Since the technique is based on low-cost hardware, it has the potential to enable well-spread traffic data collection and help improve various services in future smart cities such as transport planning, and air/ noise pollution monitoring to improve Quality of Life (QoL). 

## Figures and Tables

**Table 1:** Overview of various traffic monitoring techniques.

Technology	Concept	Examples	Advantages	Disadvantages
**Intrusive**	Installed directly into the pavement surface	Inductive loops, magnetic detectors, Micro-loop probes, pneumatic road tubes, piezoelectric and other weigh-in-motion devices [16,18]	Unresponsive to bad weather, Accurate vehicle count	Installation and maintenance need pavement cut and lane closure, expensive, large and consume much power
**Non-intrusive**	Devices mounted overhead on roadways or roadsides	Video image processing, microwave radar, laser radar, passive infrared, ultrasonic, passive acoustic array [19,20]	Vehicle speed and position information can be accurately measured, enable multiple lane monitoring	Performance affected by environmental circumstances, installation may require lane closure, expensive
**Off-roadways**	Technologies that do not require any hardware deployment under the pavement or mounted overhead/roadside	Automatic vehicle identification (AVI), Global Positioning System (GPS), mobile phones [21]	Enable high percentage of roads coverage, traffic surveillance at high accuracy	Expensive, remote sensing of aerial images for traffic monitoring is not real time, privacy concerns
**Sensor combinations**	To overcome certain limitations of individual technologies discussed above, combinations of sensors are used	Passive infrared with ultrasound, Infrared-Doppler microwave radar, Series infrared-Doppler radar-ultrasound sensors [17], Magnetic sensor with optical sensors [36]	Synergistic effect to enhance accuracy in vehicle detection	Expensive, bulky, some limitations of individual sensors and high power consumption
**Relatively low-cost devices**	Low-cost, portable, and easy-to-install technologies for real-time traffic monitoring	Continuous-wave radar [25], Computer vision low cost sensors [26], Radio-wave technologies [31,32,33,34]	Relatively less expensive than other sophisticated devices, easy to install	Specialised hardware and procedures required, limited computation capability for large dataset analysis, privacy concerns, and unsuitability for crowdsourcing applications

**Table 2:** Threshold rules for vehicle identification using Heisenbergstrasse data.

Vehicle	Threshold (in dBm)
Cars	≥611
Bicycles	<611

**Table 3:** Threshold rules for vehicle identification using Steinfurter Straße data.

Vehicle	Threshold (in dBm)
Trucks	>1849
Cars	≥357.5 &≤1849
Bicycles	<357.5

**Table 4:** Number of vehicles detected by the algorithm, according to vehicle type and classification technique for Heisenbergstrasse.

Vehicle Type	Classification Technique				Ground Truth
	Time Window	Max. RSSI	Time Window & RSSI	k-NN	
Cars	176	177	104	195	182
Bicycles	510	371	252	468	467

**Table 5:** Number of vehicles detected by the algorithm, according to vehicle type and classification technique for Steinfurter Straße.

Vehicle Type	Classification Technique				Ground Truth
	Time Window	Max. RSSI	Time Window & RSSI	k-NN	
Trucks	64	45	16	45	45
Cars	826	842	495	1004	1000
Bicycles	297	31	29	138	66

**Table 6:** Precision, Recall and F Measure using Heisenbergstrasse data (tp indicates the number of true positives).

Classification Technique	Vehicle Type	Precision	Recall	F Measure
Time window	Car (tp = 61)	0.3465	0.3351	0.3407
	Bicycle (tp = 40)	0.0784	0.0856	0.08188
Max. RSSI	Car (tp = 64)	0.3615	0.3516	0.3565
	Bicycle (tp = 47)	0.1266	0.1006	0.1121
Time window & Max.RSSI	Car (tp = 45)	0.4326	0.2472	0.3146
	Bicycle (tp = 24)	0.0952	0.0513	0.0667
k-Nearest Neighbour	Car (tp = 182)	0.934	1	0.9658
	Bicycle (tp = 467)	0.997	1	0.9984

**Table 7:** Precision, Recall and F Measure using Steinfurter Straße data (tp indicates the number of true positives).

Classification Technique	Vehicle Type	Precision	Recall	F Measure
Time window	Truck (tp = 21)	0.3281	0.4666	0.3853
	Car (tp = 425)	0.514	0.425	0.465
	Bicycle (tp = 2)	0.0067	0.0303	0.0110
Max. RSSI	Truck (tp = 15)	0.3333	0.3333	0.3333
	Car (tp = 451)	0.5356	0.451	0.4896
	Bicycle (tp = 0)	0	0	0
Time window & Max.RSSI	Truck (tp = 7)	0.4375	0.1555	0.2295
	Car (tp = 249)	0.5030	0.2490	0.3331
	Bicycle (tp = 1)	0.0344	0.0151	0.0210
k-Nearest Neighbour	Truck (tp = 42)	0.9333	0.9333	0.933
	Car (tp = 1000)	0.9960	0.9860	0.9909
	Bicycle (tp = 47)	0.3405	0.7121	0.4607

**Table 8:** Comparison of various traffic monitoring techniques to the proposed method.

Technology	High Spatial Coverage	Insensitive to Weather	Low-Cost	Compact	For Crowdsourcing	Privacy Preserving
Inductive loop [15,18]	**✗**	**✓**	**✗**	**✗**	**✗**	**✓**
Microwave radar [46]	**✗**	**✗**	**✗**	**✗**	**✗**	**✓**
Acoustic [15]	**✗**	**✓**	**✗**	**✗**	**✗**	**✓**
Magnetometer [24]	**✗**	**✗**	**✗**	**✗**	**✗**	**✓**
Infrared [17]	**✗**	**✗**	**✗**	**✗**	**✗**	**✓**
Aerial/Satellite Imaging/GPS [22]	**✓**	**✗**	**✗**	**✗**	**✓**	**✗**
Ultrasonic [20]	**✗**	**✗**	**✗**	**✗**	**✗**	**✓**
VIP (Video image processor) [19]	**✗**	**✗**	**✗**	**✗**	**✗**	**✗**
RFID (Radio-frequency identification) [47]	**✗**	**✓**	**✗**	**✗**	**✗**	**✗**
Relatively low-cost devices
Continuous-wave radar [25]	**✗**	**✓**	**✓**	**✗**	**✗**	**✓**
Computer vision [26]	**✗**	**✗**	**✓**	**✗**	**✓**	**✗**
WiFi [31]	**✓**	**✓**	**✗**	**✓**	**✓**	**✓**
Bluetooth based [35]	**✓**	**✓**	**✓**	**✓**	**✗**	**✗**
**Our Method**	**✓**	**✓**	**✓**	**✓**	**✓**	**✓**

## Abbreviations

The following abbreviations are used in this manuscript:

AVI	Automatic Vehicle Identification
CSI	Channel State Information
DSP	Digital Signal Processor
GPS	Global Positioning System
ICT	Information and Communication Technology
IoT	Internet of Things
LQI	Link Quality Indicator
MAG	Magnetometer
$NO_{x}$	Nitrogen oxides
PM	Particulate Matter
QoL	Quality of Life
RSSI	Received Signal Strength Indication
UHI	Urban Heat Islands
